# Walter Walker, M.D.

**Published:** 1868-11-15

**Authors:** 


### WALTER WALKER, M.D.— OBITUARY.

[From the Philadelphia Medical and Surgical Reporter.]

Died, September 10, 1868, in the city of Chicago, of gastro-enteritis, WALTER WALKER, M.D., of Rochester, New York, aged 31 years and 10 months.

The deceased was a graduate of Jefferson Medical College, and a most accomplished gentleman.

His mind was eminently practical, and for years he labored perseveringly and successfully to elevate the standard of ophthalmic and aural surgery to the highest point, in the Northwest.

Several surgical instruments invented by him have grown into common use, and his active mind was constantly engaged in contemplating improvements. In fact, he never seemed so well satisfied with himself as when earnestly laboring to benefit that branch of surgery to which he was devoting his life.

Kind and courteous to all, he was really and truly a Christian gentleman: earnest and persevering in the pursuit of medical knowledge, he was a sincere and blessed help to the afflicted.

He leaves a wife and three children. May God help them to believe that the terrible darkness that follows the death of such a husband and father, is indeed lessened by the vivid halo of an untarnished name—the truest and noblest heritage that a man can leave to his wife and little ones.

And when they come to stand by his (now distant) grave, may God in his goodness, soothe their bitter agony with the tender consolation, that this is the grave of a father and husband, who died young in years, but old in all that makes life worth living for; because he was true to himself, true to his family, true to his friends, and true to his God.

CHICAGO, *Sept.*, 1868. J. D. M. C.

